# Neuroprotective Effect of Puerarin on Glutamate-Induced Cytotoxicity in Differentiated Y-79 Cells via Inhibition of ROS Generation and Ca^2+^ Influx

**DOI:** 10.3390/ijms17071109

**Published:** 2016-07-11

**Authors:** Ke Wang, Xue Zhu, Kai Zhang, Zhifeng Wu, Song Sun, Fanfan Zhou, Ling Zhu

**Affiliations:** 1 Key Laboratory of Nuclear Medicine, Ministry of Health, Jiangsu Key Laboratory of Molecular Nuclear Medicine, Jiangsu Institute of Nuclear Medicine, Wuxi 214063, China; wangke@jsinm.org (K.W.); zhuxue@jsinm.org (X.Z.); zhangkai@jsinm.org (K.Z.); 2 Department of Ophthalmology, Wuxi No.2 People’s Hospital, Nanjing Medical University, Wuxi 214002, China; 3 Faculty of Pharmacy, University of Sydney, Sydney, NSW 2006, Australia; fanfan.zhou@sydney.edu.au; 4 Save Sight Institute, University of Sydney, Sydney, NSW 2000, Australia; ling.zhu@sydney.edu.au

**Keywords:** glutamate toxicity, retinal degenerative diseases, Y-79 cells, puerarin

## Abstract

Glutamate toxicity is estimated to be the key cause of photoreceptor degeneration in the pathogenesis of retinal degenerative diseases. Oxidative stress and Ca^2+^ influx induced by glutamate are responsible for the apoptosis process of photoreceptor degeneration. Puerarin, a primary component of Kudzu root, has been widely used in the clinical treatment of retinal degenerative diseases in China for decades; however, the detailed molecular mechanism underlying this effect remains unclear. In this study, the neuroprotective effect of puerarin against glutamate-induced cytotoxicity in the differentiated Y-79 cells was first investigated through cytotoxicity assay. Then the molecular mechanism of this effect regarding anti-oxidative stress and Ca^2+^ hemostasis was further explored with indirect immunofluorescence, flow cytometric analysis and western blot analysis. Our study showed that glutamate induced cell viability loss, excessive reactive oxygen species (ROS) generation, calcium overload and up-regulated cell apoptosis in differentiated Y-79 cells, which effect was significantly attenuated with the pre-treatment of puerarin in a dose-dependent manner. Furthermore, our data indicated that the neuroprotective effect of puerarin was potentially mediated through the inhibition of glutamate-induced activation of mitochondrial-dependent signaling pathway and calmodulin-dependent protein kinase II (CaMKII)-dependent apoptosis signal-regulating kinase 1(ASK-1)/c-Jun N-terminal kinase (JNK)/p38 signaling pathway. The present study supports the notion that puerarin may be a promising neuroprotective agent in the prevention of retinal degenerative diseases.

## 1. Introduction

Photoreceptor degeneration is the hallmark of many retinal degenerative diseases like the Age-related macular degeneration (AMD) and Retinitis pigmentosa (RP), which eventually leads to an irreversible loss of vision [1,2]. The cause of photoreceptor cell death is complex, such as genetic mutation and prolonged light exposure or excitotoxicity [3]. Glutamate is the main excitatory amino acid neurotransmitter in the retina and mediates the essential physiological process of visual signal transmission [4]. In the outer retina, glutamate is continuously released by both types of photoreceptor (rods and cones) and used to transmit signals to the next order neuron in the chain [5]. In the process of retinal degenerative disorders, glutamate-transporter dysfunction plays an important role in the overall pathologic manifestation of the diseases [6]. The excessive accumulation of glutamate induces over-stimulation of *N*-methyl-d-aspartate (NMDA) receptors, calcium overload and oxidative stress, which subsequently leads to neuronal injury followed by the activation of a sequence of apoptotic cascades, and results in DNA damage and apoptosis [7,8]. Human retinoblastoma Y-79 cells are derived from a multi-potential stem cell of neural retina. Following treatment with the differentiating agents of laminin and sodium butyrate, Y-79 cells can be differentiated to display prototypical properties and characteristics of photoreceptors, so they are widely used as the in vitro model to study the molecular events of photoreceptors [9,10]. In our study, we also adopt the differentiated Y-79 cells to investigate the neuroprotective effect of puerarin against glutamate-injured cytotoxicity.

Current therapeutic strategies for retinal degenerative diseases include orthodox medicines and laser treatment; however, the existing regimen is generally unsatisfied due to its short-term effect [11,12]. A rich literature including ancient Chinese medical records and modern Chinese studies in the past decades, demonstrated the success of traditional Chinese medicines in treating ocular diseases, in particular retinal degenerative diseases [13,14]. Kudzu root (Gegen in Chinese) is one of the earliest herbal medicines utilized to treat a wide range of ocular diseases in ancient China [15]. Puerarin, one of three major isoflavonoid compounds of Kudzu root, has been widely used in the clinical treatment of degenerative diseases in China for decades, due to its abilities to inhibit calcium influx, improve microcirculation, scavenge oxygen free radicals and so on [16]. Notably, puerarin was also used in the treatment of a number of retinal degenerative diseases such as AMD and RP in China, concomitantly with other drugs. This is possibly due to its protective effect against neuronal damage [15,17]. However, the detailed mechanism of such neuroprotective effect of puerarin was not fully understood.

In this study, we investigated the underlying mechanism of the neuroprotective effect of puerarin on the glutamate-induced cytotoxicity in differentiated Y-79 cells. Our findings shed new light on the therapeutic potentials of puerarin against the retinal degenerative diseases.

## 2. Results

### 2.1. The Neuroprotective Effect of Puerarin against Glutamate-Induced Cytotoxicity

**Figure 1:**
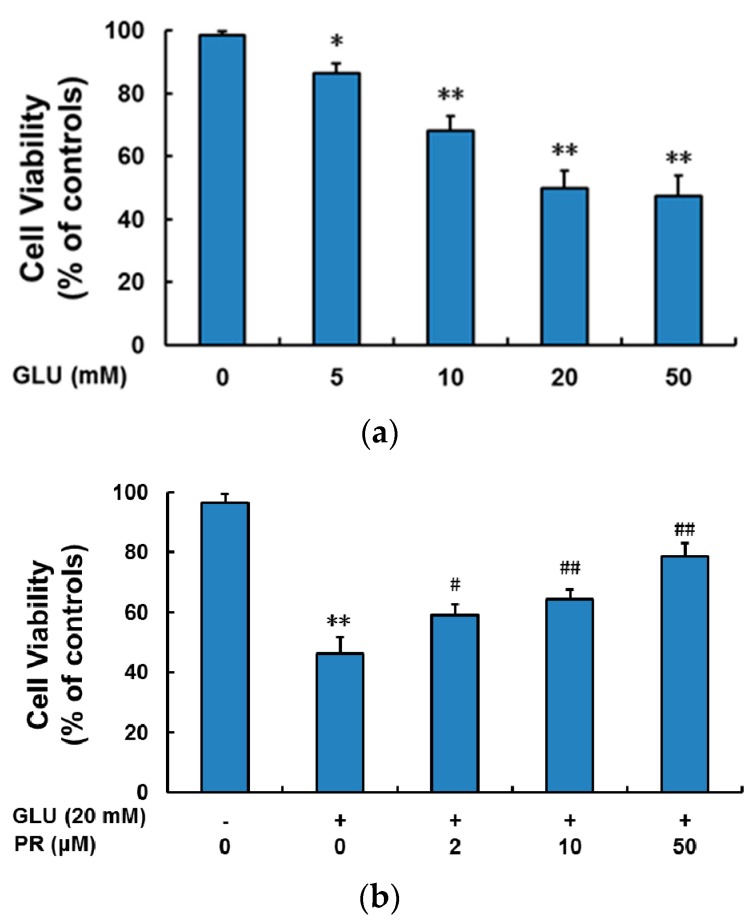
The neuroprotective effect of puerarin against the glutamate-induced cytotoxicity in the differentiated Y-79 cells. (**a**) Cells were exposed to glutamate (5–50 mM) for 24 h without puerarin pre-treatment; (**b**) cells were pre-treated with puerarin (0, 2, 10 and 50 μM) for 24 h and then exposed to 20 mM glutamate for 24 h. After treatment, cell viability was determined by MTT analysis. All data were expressed as mean ± SD of three experiments and each experiment included triplicate repeats. * *p* < 0.05, ** *p* < 0.01 vs. control group; ^#^ *p* < 0.05, ^##^ *p* < 0.01 vs. glutamate-treated group. GLU: glutamate; PR: puerarin.

Since puerarin was reported to be effective in the treatment of retinal degenerative diseases, we investigated its neuroprotective effect against glutamate-induced cytotoxicity in the differentiated Y-79 cells. First, cytotoxicity assay was adopted to establish the concentration-dependence of glutamate in inducing cytotoxicity (Figure 1a). The treatment of 20 mM glutamate for 24 h significantly induced the cell death (~50% of cell viability compared to that of control), which was consistent with previous studies in the several other neurons [18,19,20]. Hence, glutamate level at 20 mM was adopted in the following studies. Next, the differentiated Y-79 cells were pre-treated with or without puerarin at the indicated concentrations followed by glutamate and then cell viability was assessed with MTT assay. As shown in Figure 1b, pre-treatment of puerarin (2, 10 and 50 μM) significantly attenuated the glutamate-induced cell damage at a dose-dependent manner and restored the cell viability to 58.92% ± 3.71%, 64.43% ± 3.18% and 78.65% ± 4.23% of control, respectively.

### 2.2. The Neuroprotective Effect of Puerarin against Glutamate-Induced Reactive Oxygen Species (ROS) Generation

**Figure 2:**
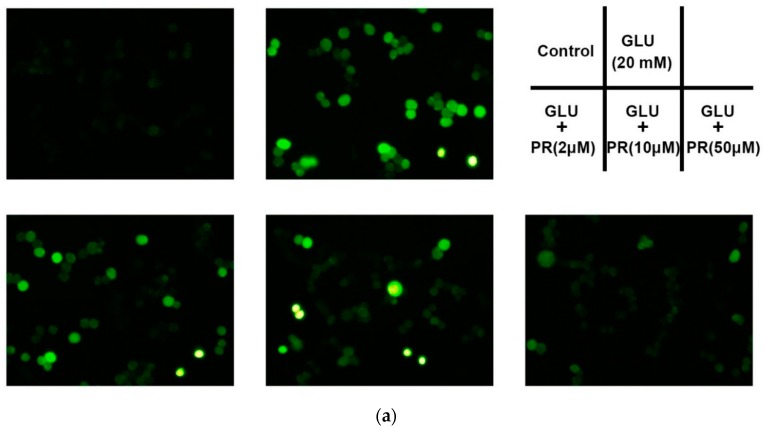

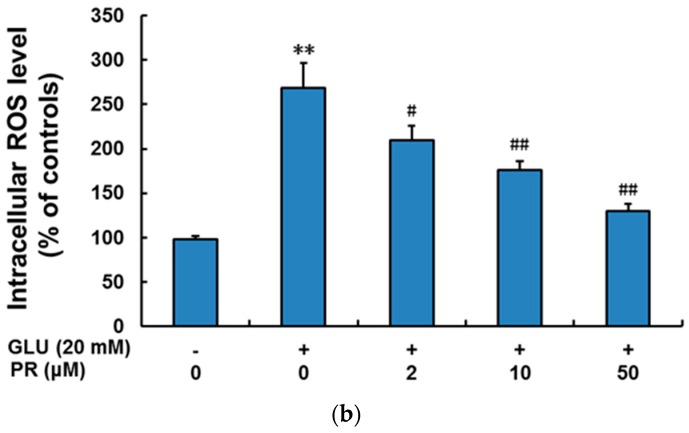
The neuroprotective effect of puerarin against the glutamate-induced reactive oxygen species (ROS) generation in the differentiated Y-79 cells. Cells were pre-treated with puerarin (0, 2, 10 and 50 μM) for 24 h and then exposed to 20 mM glutamate for 6 h. After treatment, cells were incubated with dichloro-dihydro-fluorescein diacetate (DCFH-DA) (10 μM) at 37 °C for 30 min in dark. (**a**) Representative microphotographs of fluorescence staining; (**b**) the relative fluorescence intensity was analyzed by flow cytometry. All data were expressed as mean ± SD of three experiments and each experiment included triplicate repeats. ** *p* < 0.01 vs. control group; ^#^ *p* < 0.05, ^##^ *p* < 0.01 vs. glutamate-treated group. GLU: glutamate, PR: puerarin.

Glutamate neurotoxicity can induce oxidative stress and consequently results in excessive reactive oxygen species (ROS) generation. In this study, we investigated the protective effect of puerarin against glutamate-induced ROS accumulation in the differentiated Y-79 cells. As shown in Figure 2, exposure to 20 mM glutamate for 6 h dramatically increased ROS generation from 98.37% ± 3.63% to 268.21% ± 28.15% compared to the control. However, the pre-treatment of puerarin (2, 10 and 50 μM) significantly reversed the glutamate-induced ROS generation to 209.34% ± 16.41%, 175.72% ± 10.61% and 129.35% ± 8.85% of the control, respectively.

### 2.3. The Neuroprotective Effect of Puerarin against Glutamate-Induced Ca^2+^ Influx

**Figure 3:**
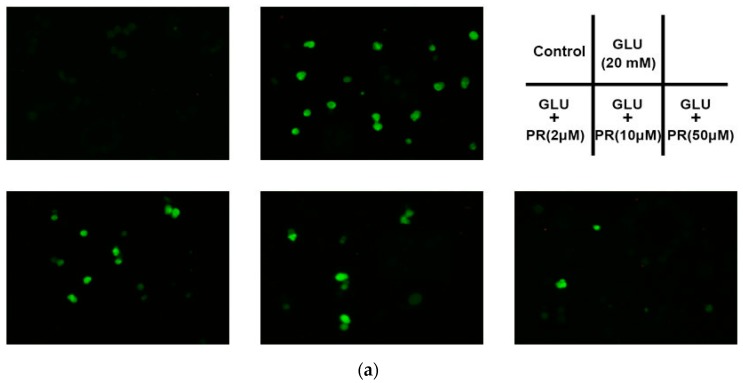

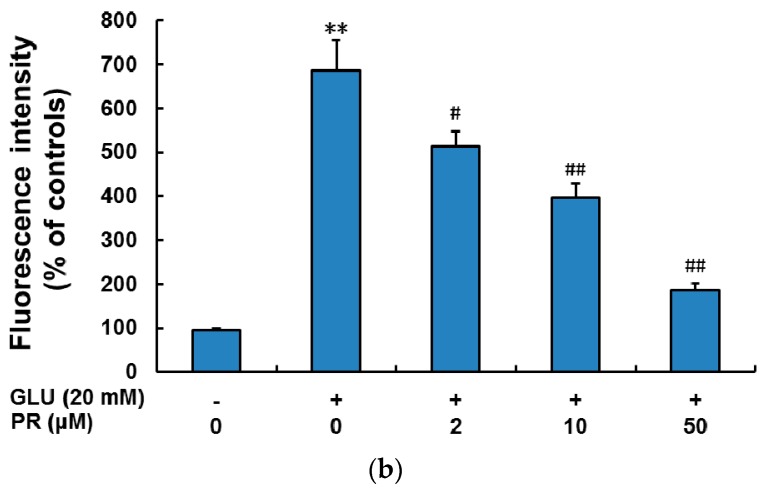
The neuroprotective effect of puerarin against the glutamate-induced Ca^2+^ influx in the differentiated Y-79 cells. Cells were pre-treated with puerarin (0, 2, 10 and 50 μM) for 24 h and then exposed to 20 mM glutamate for 12 h. After treatment, cells were incubated with Fluo-3/AM working solution at 37 °C for 30 min in dark. (**a**) Representative microphotographs of fluorescence staining; (**b**) the relative fluorescence intensity was analyzed by microplate reader. All data were expressed as mean ± SD of three experiments and each experiment included triplicate repeats. ** *p* < 0.01 vs. control group; ^#^ *p* < 0.05, ^##^ *p* < 0.01 vs. glutamate-treated group. GLU: glutamate, PR: puerarin.

Glutamate-induced Ca^2+^ overload is the major cause of neuronal death. In this study, we investigated the protective effect of puerarin against glutamate-induced Ca^2+^ influx in the differentiated Y-79 cells. As shown in Figure 3, the relative fluorescence intensity of Fluo-3/AM was increased from 95.12% ± 4.88% to 686.17% ± 68.27% after treatment with 20 mM glutamate for 12 h compared to the control. However, the pre-treatment of puerarin (2, 10 and 50 μM) pronouncedly reversed the glutamate-induced Ca^2+^ influx to 513.49% ± 34.58%, 397.29% ± 30.55% and 186.56% ± 15.84%, respectively.

### 2.4. The Neuroprotective Effect of Puerarin against Glutamate-Induced Apoptosis

**Figure 4:**
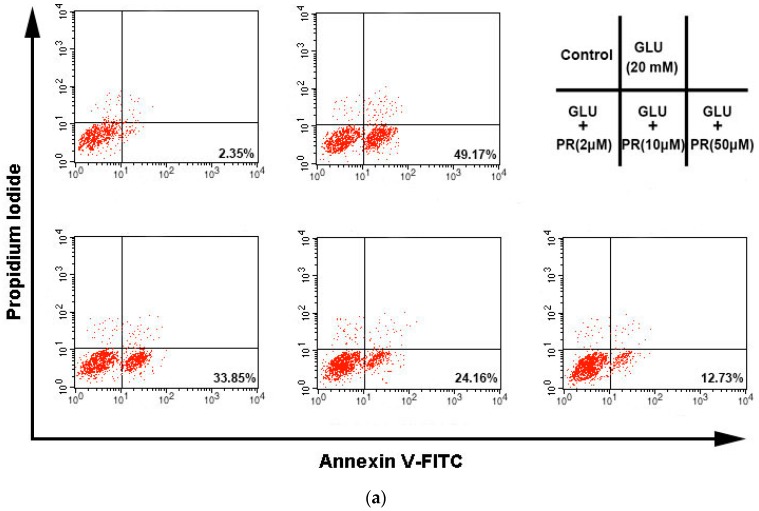

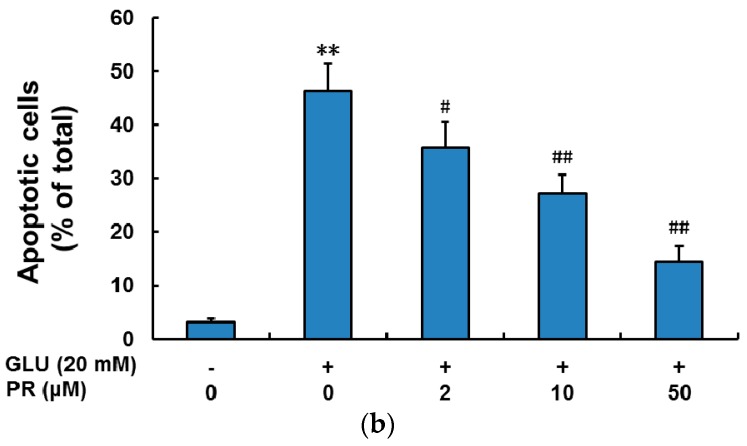
The neuroprotective effect of puerarin against glutamate-induced apoptosis in the differentiated Y-79 cells. Cells were pre-treated with puerarin (0, 2, 10 and 50 μM) for 24 h and then exposed to 20 mM glutamate for 24 h. After treatment, cells were re-suspended in 300 μL binding buffer containing 10 μL of Annexin V-FITC stock and 10 μL of propidium iodide (PI), and then incubated at room temperature for 15 min in the dark. (**a**) Flow cytometry analysis of cell apoptosis using Annexin V-FITC/PI dual-staining; (**b**) the densitometric analysis of the percentage of apoptotic cells. All data were expressed as mean ± SD of three experiments and each experiment included triplicate repeats. ** *p* < 0.01 vs. control group; ^#^ *p* < 0.05, ^##^ *p* < 0.01 vs. glutamate-treated group. GLU: glutamate, PR: puerarin.

Glutamate-induced ROS generation and Ca^2+^ influx can eventually lead to cell apoptosis. In this study, we therefore investigated the protective effect of puerarin against glutamate-induced apoptosis in the differentiated Y-79 cells. As shown in Figure 4, exposure to 20 mM glutamate for 24 h remarkably increased the apoptosis from 3.17% ± 0.68% to 46.23% ± 5.18% compared to the control. Interestingly, the pre-treatment of puerarin (2, 10 and 50 μM) potently reversed the glutamate-induced apoptosis to 35.63% ± 4.82%, 27.18% ± 3.53% and 14,45% ± 2.84%, respectively.

### 2.5. Puerarin Attenuated Glutamate-Induced Activation of Mitochondrial-Dependent Signaling Pathway

**Figure 5:**
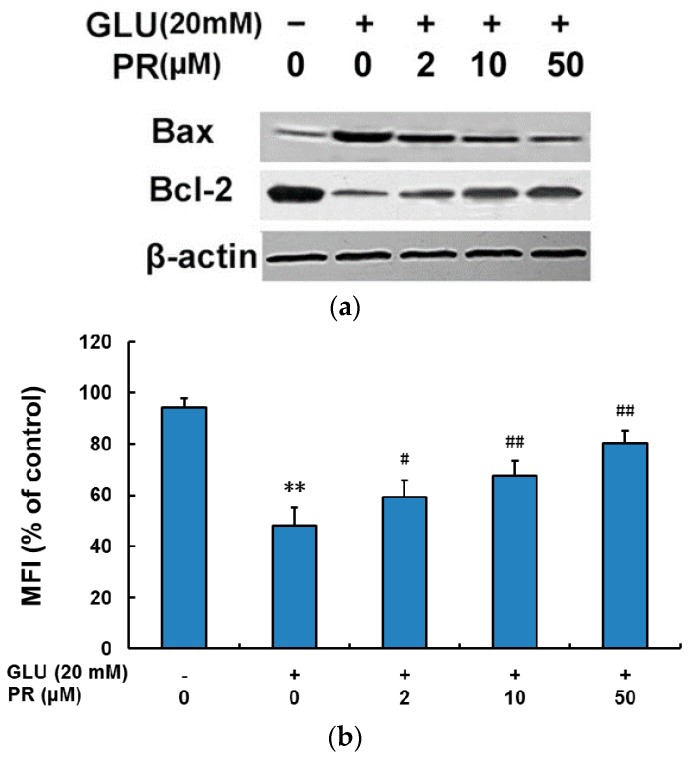

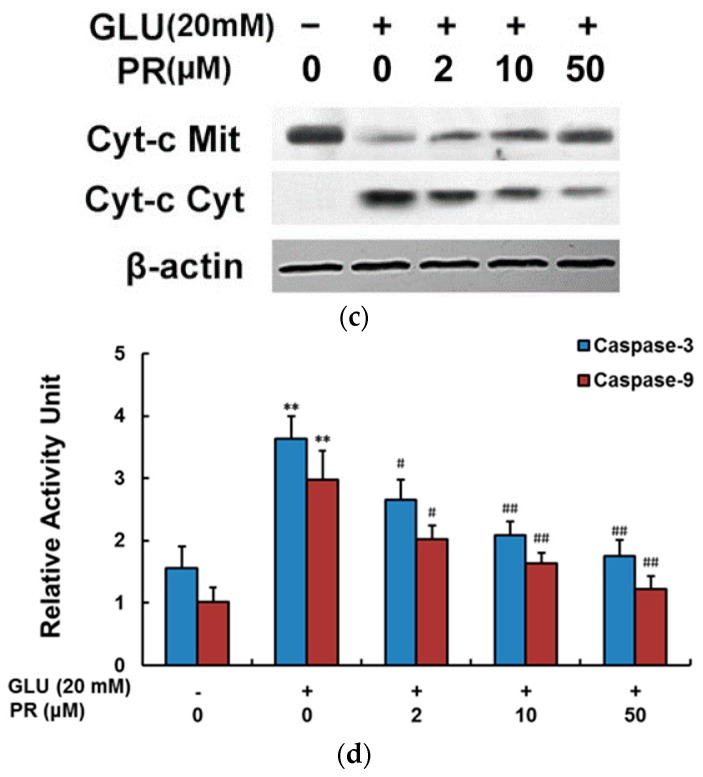
Puerarin attenuated glutamate-induced activation of mitochondrial-dependent signaling pathways. Cells were pre-treated with puerarin (0, 2, 10 and 50 μM) for 24 h and then exposed to 20 mM glutamate for 24 h. (**a**) The expression levels of Bax and Bcl-2 were determined by western blot analysis; (**b**) fluorescence ratio was used for mitochondria membrane potential (MMP) quantitative analysis; (**c**) the levels of cyto and mito cytochrome c were detected by western blot analysis; (**d**) the activities of caspase-9 and caspase-3 were determined. All data were expressed as mean ± SD of three experiments and each experiment included triplicate repeats. ** *p* < 0.01 vs. control group; ^#^ *p* < 0.05, ^##^ *p* < 0.01 vs. glutamate-treated group. GLU: glutamate, PR: puerarin.

Mitochondrial dysfunction is one of the prominent events of oxidative injury and cell apoptosis. In this study, we explored the signaling pathways involved in the neuroprotective effect of puerarin against glutamate-induced mitochondrial dysfunction. As shown in Figure 5, exposure to 20 mM glutamate for 24 h induced the up-regulation of Bax and down-regulation of Bcl-2, increase of mitochondrial membrane potential, cytochrome c release from mitochondria as well as the activation of caspase-9 and caspase-3. The pre-treatment of puerarin (2, 10 and 50 μM) effectively reversed the modulation of these signaling pathways in a dose-dependent manner.

### 2.6. Puerarin Attenuated Glutamate-Induced Activation of Calmodulin-Dependent Protein Kinase II (CaMKII)-Dependent Apoptosis Signal-Regulating Kinase 1(ASK-1)/c-Jun N-Terminal Kinase (JNK)/p38 Signaling Pathway

**Figure 6:**
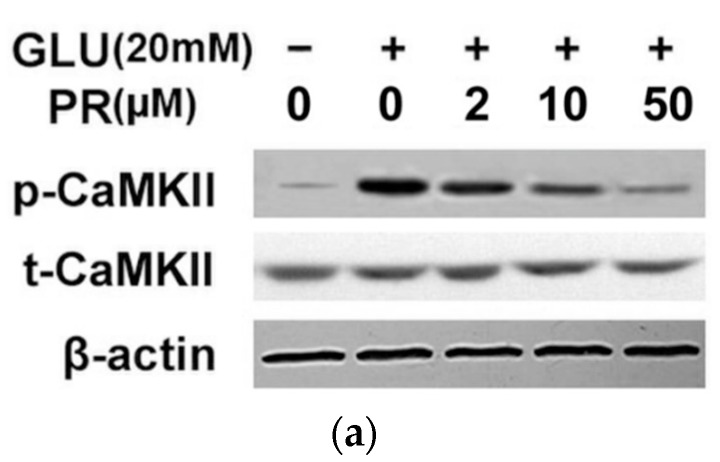

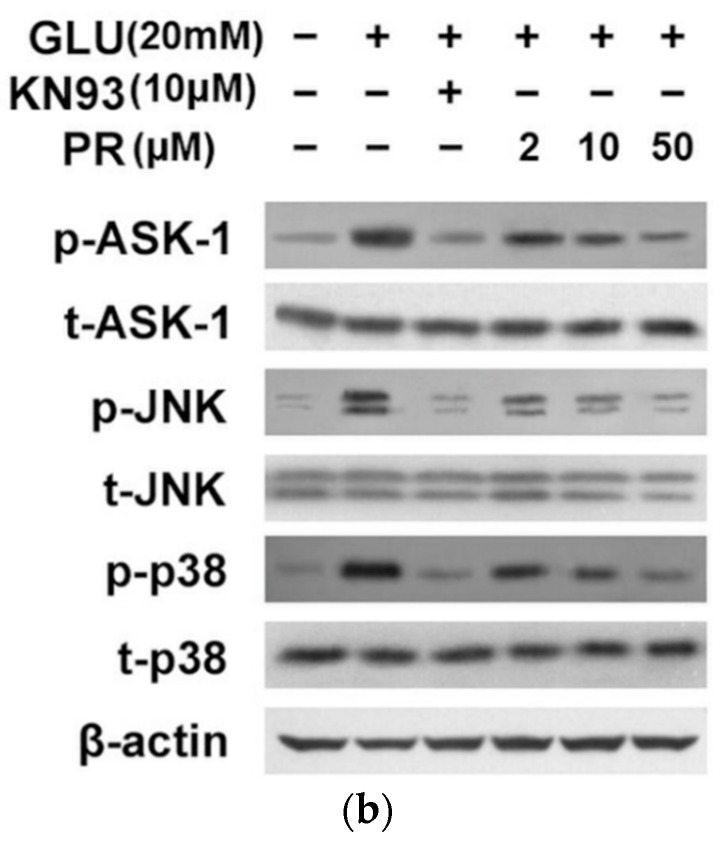
Puerarin attenuated the glutamate-induced activation of calmodulin-dependent protein kinase II (CaMKII)-dependent apoptosis signal-regulating kinase 1(ASK-1)/c-Jun N-terminal kinase (JNK)/p38 signaling pathway. Cells were pre-treated with puerarin (0, 2, 10 and 50 μM) for 24 h and then exposed to 20 mM glutamate for 24 h. (**a**,**b**) The expression levels of relative proteins were determined by western blot analysis using indicated antibodies. All data were representative of three independent experiments. The internal control was β-actin.

The glutamate-induced Ca^2+^ influx can activate calmodulin-dependent protein kinase II (CaMKII)-dependent apoptosis signal-regulating kinase 1(ASK-1)/c-Jun N-terminal kinase (JNK)/p38 MAPK signaling pathway and eventually result in Ca^2+^ overload-mediated apoptosis. In this study, we assessed the phosphorylation of CaMKII and ASK/JNK/p38 signaling cascades with the treatment of indicated drugs. As shown in Figure 6, exposure to 20 mM glutamate for 24 h significantly induced the phosphorylation of CaMKII and ASK/JNK/p38 proteins; while CaMKII inhibitor KN93 can significantly reverse the glutamate-induced phosphorylation of ASK/JNK/p38 signaling. Furthermore, the pre-treatment of puerarin (2, 10 and 50 μM) significantly reversed such effects in a dose-dependent manner.

## 3. Discussion

Accumulating evidence has postulated that glutamate toxicity is the key cause of neuronal death in retinal degenerative diseases [21]. At least, the glutamate receptor-mediated and oxidative stress-mediated toxicity are known to be responsible for the glutamate-induced toxicity in neuron injury [22,23]. Glutamate receptor-mediated toxicity is associated with the activation of NMDA and non-NMDA glutamatergic receptors and subsequent induction of calcium influx into the cells, leading to an intracellular cascade of cytotoxic events [24]. Oxidative stress-mediated toxicity is a transporter-mediated cell death that requires the cellular expression of the cystine/glutamate antiporter system. The inhibited uptake of cystine leads to marked decrease of the intracellular glutathione (GSH) levels, resulting in the induction of oxidative stress and cell death [25]. In this study, glutamate (20 mM) was used as a pathologically relevant stressor due to its direct pathological role in disease states. Then the molecular mechanism for glutamate neurotoxicity in the differentiated Y-79 cells, an approximate in vitro model of photoreceptors, was investigated. Our results showed that these two mechanisms were all involved in such a process.

Puerarin, a primary component of Kudzu root, has recently been reported to be an active neuroprotective agent in the models of neurodegenerative diseases such as Alzheimer’s disease (AD), Parkinson’s disease (PD) and Amyotrophic lateral sclerosis (ALS) [26,27]. Puerarin has also been widely used in the clinical treatment of retinal degenerative diseases in China for decades due to its neuroprotective effect. However, the detailed molecular mechanism underlying this effect remains unknown. In this study, we elucidated the molecular mechanism of puerarin against glutamate-induced cytotoxicity in differentiated Y-79 cells for the first time. Our data showed that the neuroprotective effect of puerarin targeted at oxidative stress and Ca^2+^ hemostasis. We showed that the pre-treatment of puerarin (2, 10 and 50 μM) could significantly attenuate glutamate (20 mM)-induced cell viability loss, excessive ROS generation, calcium overload and up-regulated cell apoptosis in a dose-dependent manner.

Increasing evidence has suggested that glutamate accumulation induced oxidative stress plays a key role in retinal neuron cell death [28]. ROS is the major factor of oxidative stress, the massive accumulation of which can lead to cell apoptosis by inducing mitochondrial dysfunction [29]. Therefore, this study investigated the protective effect of puerarin against the glutamate-induced ROS generation and mitochondrial dysfunction in the differentiated Y-79 cells. Our results showed that exposure to 20 μM glutamate for 24 altered the expression levels of Bcl-2 family proteins, the operation of pores in cell membrane, the release of cytochrome c from mitochondria and activation of caspases. The pre-treatment of puerarin (2, 10 and 50 μM) remarkably reversed these effects in a dose-dependent manner. Hence, our data suggested that the neuroprotection of puerarin against glutamate toxicity in the differentiated Y-79 cells was largely related to its antioxidant ability.

Compelling evidence has indicated that glutamate accumulation induced Ca^2+^ overload is another major cause of retinal neuron cell death, which also induces the formation of new ROS [30]. Ca^2+^ influx induces the activation of CaM (calmodulin) and which further stimulates CaMK (calmodulin-dependent protein kinase) phosphorylation. CaMKII is an important member of the calcium/calmodulin-activated protein kinase family, functioning in a number of neuronal synaptic stimulation [31]. Our study showed that Ca^2+^/CaMKII transcriptional pathway was activated in the glutamate-induced cell injury of the differentiated Y-79 cells, which could be attenuated by puerarin dose-dependently. Ca^2+^/CaMKII signaling pathway is involved with the regulation of ASK-1/JNK/p38 MAPKs signaling and thus, induces cell apoptosis [32]. We showed that exposure to 20 mM glutamate for 24 h significantly induced the phosphorylation of CaMKII and ASK-1/JNK/p38 proteins and CaMKII inhibitor KN93 could significantly reverse the glutamate-induced ASK/JNK/p38 signaling cascade. Furthermore, the pre-treatment of puerarin potently reversed this effect in a dose-dependent manner. Hence, our data suggested that to inactivate CaMKII-dependent ASK-1/JNK/p38 signaling pathway was another mechanism underlying the neuroprotective effect of puerarin against glutamate toxicity in the differentiated Y-79 cells.

## 4. Materials and Methods

### 4.1. Materials

Puerarin (purity > 98%) purchased from National Institute for the Control of Pharmaceutical and Biological Products (Beijing, China) was used in this study. l-glutamate, 3-(4,5-dimethylthiazol-2-yl)-2,5-diphenyltetrazolium bromide (MTT), Fluo-3/AM and Rhodamine123 were obtained from Sigma-Aldrich (St. Louis, MO, USA). ROS activity assay kit was obtained from Abnova (Walnut, CA, USA). Apoptosis detection kit was obtained from BD Biosciences (Franklin Lakes, NJ, USA). Antibodies were obtained from Santa Cruz Biotechnology (Dallas, TX, USA) and Cell Signaling Technology (Beverly, MA, USA). Caspase-3,9 fluorometric assay kits were obtained from BioVision (Milpitas, CA, USA). All other chemicals and reagents were purchased from Beyotime Biotech (Nangtong, China).

### 4.2. Cell Culture

The human retinoblastoma cell line Y-79 was obtained from American Type Culture Collection (ATCC, Manassas, VA, USA). The cells were verified by the ATCC Cell Line Authentication Service (Promega, Madison, WI, USA). Y-79 cells were cultured in RPMI-1640 medium containing 10% fetal bovine serum (FBS), 1250 μg of fungizone, 100 U/mL penicillin and 100 μg/mL streptomycin (Life Technologies, Carlsbad, CA, USA) at 37 °C (95% air, 5% CO_2_). Cells were plated in poly-d-lysine-coated culture plate with laminin (10 μg/mL) for 24 h, induced to neuronal differentiation with sodium butyrate (1 mM) for 3 days, and then used for the following experiments.

### 4.3. Cell Viability Assay

Cell viability assay was conducted using MTT method [33]. Differentiated Y-79 cells seeded in 96-well culture plate were incubated with drugs for the indicated time. After treatment, MTT (10 μL, 1 mg/mL) was added to each well and incubated for an additional 4 h at 37 °C. The culture medium was then aspirated and DMSO (100 μL) was added to dissolve the insoluble dark blue formazan crystals. The absorbance was measured at the wavelength of 490 nm with a microplate reader (Molecular Devices, Sunnyvale, CA, USA). Cell viability was expressed as a percentage with the control group as 100%.

### 4.4. ROS Activity Assay

Intracellular ROS generation measurement was conducted by flow cytometry using dichloro-dihydro-fluorescein diacetate (DCFH-DA) staining [34]. DCFH-DA is a well-established compound to detect and quantify intracellular produced ROS. After treatment, cells were re-suspended in DCFH-DA (10 μM) working solution at 37 °C for 30 min in dark and washed twice with PBS. Intracellular ROS generation was taken by fluorescence microscope (Leica Microsystems, Wetzlar, Germany). The fluorescence intensity was analyzed by flow cytometry. ROS activity was expressed as a percentage with the control group as 100%.

### 4.5. Intracellular Ca^2+^ Measurement

Intracellular calcium concentration measurement was conducted using fluorescent dye Fluo-3/AM staining [35]. After treatment, cells were re-suspended in Fluo-3/AM working solution (5 μM) at 37 °C for 30 min in the dark and washed twice with PBS. After that, cells were incubated in culture medium for an another 20 min in the dark. Intracellular Ca^2+^ influx was taken by fluorescence microscope (Leica Microsystems). The fluorescence intensity was analyzed by microplate reader (Molecular Devices). Intracellular Ca^2+^ level was expressed as a percentage with the control group as 100%.

### 4.6. Cell Apoptosis Assay

Cell apoptosis was determined by flow cytometric analysis using Annexin V-FITC and PI apoptosis kit (BD Biosciences, Franklin Lakes, NJ, USA). After treatment, cells were re-suspended in binding buffer, stained with 10 μL of Annexin V-FITC and 10 μL of PI, and then incubated at room temperature for 15 min in the dark. The stained cells were analyzed by a flow cytometer (BD Biosciences). The apoptotic cells were expressed as a percentage of the total number of cells.

### 4.7. Mitochondria Membrane Potential (MMP) Measurement

Mitochondria membrane potential (MMP) was detected by flow cytometry using fluorescent dye Rhodamine123 [36]. After treatment, cells were re-suspended in Rhodamine123 (5 μM) working solution at 37 °C for 30 min in the dark and washed twice with PBS. Analysis was performed by flow cytometry at wavelengths of 490 nm (excitation)/530 nm (emission) and 530 nm (excitation)/590 nm (emission). The alternation of MMP level was calculated by the changes in the ratio between the measurement at wavelengths of 590 nm (red) and 530 nm (green) fluorescence intensities.

### 4.8. Cytochrome c Release Measurement

The cytosol and mitochondrial fractions were respectively collected as described previously to measure the release of cytochrome c [37]. After treatment, mitochondrial and cytosolic fractions were extracted from the cells using Apo Alert Cell Fractionation Kit (Clontech, Mountain View, CA, USA) according to the manufacturer’s instructions. Cytochrome c expression was analyzed using a monoclonal antibody through western blot analysis.

### 4.9. Western Blot Analysis

Western blot analysis was conducted as previously described [38]. After treatment, cells were collected and lysed in RIPA buffer. Protein concentration was quantified by Bradford assay [39]. The protein samples were electrophoresed on 12% SDS-polyacrylamide gel (SDS-PAGE) and transferred to polyvinylidene fluoride (PVDF) membranes. Each membrane was blocked with skim milk in TBST buffer for 1 h and then probed with primary antibody at 4 °C overnight. After washed with TBST for three times, the membrane was incubated with secondary antibody for 1 h at room temperature. The membrane was visualized using the enhanced chemiluminescence (ECL) western detection kit (Beyotime, Nantong, China). In addition, re-probing method was used in a single membrane for investigating more than one protein with Western ReProbe™ (G-Biosciences, St. Louis, MO, USA).

### 4.10. Caspase Activity Assay

Caspase activity assay was conducted using fluorogenic caspase substrate. After treatment, cells were collected and lysed in assay buffer. Aliquots of crude cell lysate were incubated with caspase substrate at 37 °C for 30 min in the dark. The caspase activity was determined by measuring the relative fluorescence intensity at 505 nM following excitation at 400 nm using a spectrofluorometer (Molecular Devices). Caspase activity was expressed as a percentage with the control group as 100%.

### 4.11. Statistical Analysis

Statistical analyses were conducted with the SPSS 16.0 software. Data were presented as mean ± SD. Statistical significance was analyzed by Student’s *t*-test and One-way ANOVA. *p* value smaller than 0.05 was considered significant.

## 5. Conclusions

In summary, our study showed that glutamate induced cell viability loss, excessive ROS generation, calcium overload and up-regulated cell apoptosis in Y-79 cells, which effect was significantly attenuated with the pre-treatment of puerarin in a dose-dependent manner. Furthermore, our data indicated that the neuroprotective effect of puerarin was potentially mediated through the inhibition of glutamate-induced activation of mitochondrial-dependent signaling pathway and CaMKII-dependent ASK-1/JNK/p38 signaling pathway. The present study supports the notion that puerarin may be a promising neuroprotective agent in the prevention of retinal degenerative diseases.

## References

[1] Curcio C.A., Medeiros N.E., Millican C.L. (1996). Photoreceptor loss in age-related macular degeneration. Investig. Ophthalmol. Vis. Sci..

[2] Bovolenta P., Cisneros E. (2009). Retinitis pigmentosa: Cone photoreceptors starving to death. Nat. Neurosci..

[3] Wright A.F., Chakarova C.F., Abd El-Aziz M.M., Bhattacharya S.S. (2010). Photoreceptor degeneration: Genetic and mechanistic dissection of a complex trait. Nat. Rev. Genet..

[4] Ishikawa M. (2013). Abnormalities in glutamate metabolism and excitotoxicity in the retinal diseases. Scientifica.

[5] Delyfer M.N., Forster V., Neveux N., Picaud S., Leveillard T., Sahel J.A. (2005). Evidence for glutamate-mediated excitotoxic mechanisms during photoreceptor degeneration in the rd1 mouse retina. Mol. Vis..

[6] Greenamyre J.T. (1986). The role of glutamate in neurotransmission and in neurologic disease. Arch. Neurol..

[7] Reynolds I.J., Hastings T.G. (1995). Glutamate induces the production of reactive oxygen species in cultured forebrain neurons following NMDA receptor activation. J. Neurosci..

[8] Kruman I.I., Mattson M.P. (1999). Pivotal role of mitochondrial calcium uptake in neural cell apoptosis and necrosis. J. Neurochem..

[9] Chader G.J. (1987). Multipotential differentiation of human Y-79 retinoblastoma cells in attachment culture. Cell Differ..

[10] Albini A., Noonan D.M., Melchiori A., Fassina G.F., Percario M., Gentleman S., Toffenetti J., Chader G.J. (1992). Laminin-induced retinoblastoma cell differentiation: Possible involvement of a 100-kDa cell-surface laminin-binding protein. Proc. Natl. Acad. Sci. USA.

[11] Nowak J.Z. (2006). Age-related macular degeneration (AMD): Pathogenesis and therapy. Pharmacol. Rep..

[12] Delyfer M.N., Leveillard T., Mohand-Said S., Hicks D., Picaud S., Sahel J.A. (2004). Inherited retinal degenerations: Therapeutic prospects. Biol. Cell.

[13] Wilkinson J.T., Fraunfelder F.W. (2011). Use of herbal medicines and nutritional supplements in ocular disorders: An evidence-based review. Drugs.

[14] Wang L., Wang N., Tan H.Y., Zhang Y., Feng Y. (2015). Protective effect of a Chinese Medicine formula He-Ying-Qing-Re Formula on diabetic retinopathy. J. Ethnopharmacol..

[15] Xuan B., Zhou Y.H., Yang R.L., Li N., Min Z.D., Chiou G.C. (1999). Improvement of ocular blood flow and retinal functions with puerarin analogs. J. Ocul. Pharmacol. Ther..

[16] Zhu X., Wang K., Zhang K., Lin X., Zhu L., Zhou F. (2016). Puerarin Protects Human Neuroblastoma SH-SY5Y Cells against Glutamate-Induced Oxidative Stress and Mitochondrial Dysfunction. J. Biochem. Mol. Toxicol..

[17] Zhu X., Xie M., Wang K., Zhang K., Gao Y., Zhu L., Zhou F. (2014). The effect of puerarin against IL-1β-mediated leukostasis and apoptosis in retinal capillary endothelial cells (TR-iBRB2). Mol. Vis..

[18] Ding Z.J., Chen X., Tang X.X., Wang X., Song Y.L., Chen X.D., Mi W.J., Wang J., Lin Y., Chen F.Q. (2015). Calpain inhibitor PD150606 attenuates glutamate induced spiral ganglion neuron apoptosis through apoptosis inducing factor pathway in vitro. PLoS ONE.

[19] Chang C.-H., Chen H.-X., Yü G., Peng C.-C., Peng R.Y. (2014). Curcumin-protected PC12 cells against glutamate-induced oxidative toxicity. Food Technol. Biotechnol..

[20] Nampoothiri M., Reddy N.D., John J., Kumar N., Kutty Nampurath G., Rao Chamallamudi M. (2014). Insulin blocks glutamate-induced neurotoxicity in differentiated SH-SY5Y neuronal cells. Behav. Neurol..

[21] Schmidt K.G., Bergert H., Funk R.H.W. (2008). Neurodegenerative Diseases of the Retina and Potential for Protection and Recovery. Curr. Neuropharmacol..

[22] Kanki R., Nakamizo T., Yamashita H., Kihara T., Sawada H., Uemura K., Kawamata J., Shibasaki H., Akaike A., Shimohama S. (2004). Effects of mitochondrial dysfunction on glutamate receptor-mediated neurotoxicity in cultured rat spinal motor neurons. Brain Res..

[23] Chen J., Chua K.W., Chua C.C., Yu H., Pei A., Chua B.H., Hamdy R.C., Xu X., Liu C.F. (2011). Antioxidant activity of 7,8-dihydroxyflavone provides neuroprotection against glutamate-induced toxicity. Neurosci. Lett..

[24] Michaels R.L., Rothman S.M. (1990). Glutamate neurotoxicity in vitro: Antagonist pharmacology and intracellular calcium concentrations. J. Neurosci..

[25] Bridges R.J., Natale N.R., Patel S.A. (2012). System x_c_^−^ cystine/glutamate antiporter: An update on molecular pharmacology and roles within the CNS. Br. J. Pharmacol..

[26] Zhou Y.-X., Zhang H., Peng C. (2013). Puerarin: A Review of Pharmacological Effects. Phytother. Res..

[27] Tian F., Xu L.H., Wang B., Tian L.J., Ji X.L. (2015). The neuroprotective mechanism of puerarin in the treatment of acute spinal ischemia-reperfusion injury is linked to cyclin-dependent kinase 5. Neurosci. Lett..

[28] Nakayama M., Aihara M., Chen Y.N., Araie M., Tomita-Yokotani K., Iwashina T. (2011). Neuroprotective effects of flavonoids on hypoxia-, glutamate-, and oxidative stress-induced retinal ganglion cell death. Mol. Vis..

[29] Cui H., Kong Y., Zhang H. (2012). Oxidative Stress, Mitochondrial Dysfunction, and Aging. J. Signal. Transduct..

[30] Sucher N.J., Lipton S.A., Dreyer E.B. (1997). Molecular basis of glutamate toxicity in retinal ganglion cells. Vis. Res..

[31] Timmins J.M., Ozcan L., Seimon T.A., Li G., Malagelada C., Backs J., Backs T., Bassel-Duby R., Olson E.N., Anderson M.E. (2009). Calcium/calmodulin-dependent protein kinase II links ER stress with Fas and mitochondrial apoptosis pathways. J. Clin. Investig..

[32] Brnjic S., Olofsson M.H., Havelka A.M., Linder S. (2010). Chemical biology suggests a role for calcium signaling in mediating sustained JNK activation during apoptosis. Mol. Biosyst..

[33] Van Meerloo J., Kaspers G.J., Cloos J. (2011). Cell sensitivity assays: The MTT assay. Methods Mol. Biol..

[34] Massaro M., Basta G., Lazzerini G., Carluccio M.A., Bosetti F., Solaini G., Visioli F., Paolicchi A., de Caterina R. (2002). Quenching of intracellular ROS generation as a mechanism for oleate-induced reduction of endothelial activation and early atherogenesis. Thromb. Haemost..

[35] Ma S., Liu H., Jiao H., Wang L., Chen L., Liang J., Zhao M., Zhang X. (2012). Neuroprotective effect of ginkgolide K on glutamate-induced cytotoxicity in PC 12 cells via inhibition of ROS generation and Ca^2+^ influx. Neurotoxicology.

[36] Wang K., Zhu X., Zhang K., Zhu L., Zhou F. (2014). Investigation of gallic acid induced anticancer effect in human breast carcinoma MCF-7 cells. J. Biochem. Mol. Toxicol..

[37] Zhu X., Wang K., Zhang K., Zhu L., Zhou F. (2014). Ziyuglycoside II induces cell cycle arrest and apoptosis through activation of ROS/JNK pathway in human breast cancer cells. Toxicol. Lett..

[38] Mahmood T., Yang P.-C. (2012). Western Blot: Technique, Theory, and Trouble Shooting. N. Am. J. Med. Sci..

[39] Kruger N.J. (2009). The Bradford method for protein quantitation. The Protein Protocols Handbook.

